# CXCL13 Is Involved in the Lipopolysaccharide-Induced Hyperpermeability of Umbilical Vein Endothelial Cells

**DOI:** 10.1007/s10753-020-01253-6

**Published:** 2020-06-04

**Authors:** Wen Chen, Yi Wang, Ting Zhou, Yuansheng Xu, Jianwei Zhan, Jinhong Wu

**Affiliations:** 1 Department of General Practice, Hangzhou First People’s Hospital affiliated to Zhejiang University School of Medicine, Hangzhou, Zhejiang China; 2 Department of Emergency, Hangzhou First People’s Hospital affiliated to Zhejiang University School of Medicine, Hangzhou, Zhejiang China

**Keywords:** CXCL13, LPS, Permeability, p38

## Abstract

Sepsis is a disease that is characterized by a severe systemic inflammatory response to microbial infection and lipopolysaccharide (LPS) and is a well-known inducer of sepsis, as well as endothelial cell hyperpermeability. In the present study, we confirm the elevation of CXC chemokine ligand 13 (CXCL13) in sepsis patients. We also show that LPS exposure increases the release of CXCL13, as well as the mRNA and protein expression of CXCL13 and its receptor, CXC chemokine receptor 5 (CXCR5) in human umbilical vein endothelial cells (HUVECs) in a dose- and time-dependent manner. We also examined the effects of CXCL13 knockdown on LPS-mediated endothelial hyperpermeability and tight junction (TJ) protein expression in HUVECs. Our results show that HUVECs exposed to LPS result in a significant decrease in transendothelial electrical resistance (TER) and TJ protein (Zonula occluden-1, occludin, and claudin-4) expression, and a notable increase in fluorescein isothiocyanate (FITC)-dextran flux and p38 phosphorylation, which was partially reversed by CXCL13 knockdown. Recombinant CXCL13 treatment had a similar effect as LPS exposure, which was attenuated by a p38 inhibitor, SB203580. Moreover, the CXCL13-neutralizing antibody significantly increased the survival rate of LPS-induced sepsis mice. Collectively, our results show that CXCL13 plays a key role in LPS-induced endothelium hyperpermeability *via* regulating p38 signaling and suggests that therapeutically targeting CXCL13 may be beneficial for the treatment of sepsis.

## INTRODUCTION

Sepsis is a disease characterized by a severe systemic inflammatory response to microbial infection [1]. The disease affects more than 18 million people every year [2], and despite intense efforts, poor prognosis is observed in patients with severe sepsis, with 40–60% a mortality rate [3, 4]. Most of the septic response is caused by endotoxin or lipopolysaccharide (LPS), the cell wall component of bacteria. Microvascular dysfunction is regarded as a hallmark of sepsis [5]. Inflammatory mediators and free radicals, produced by the septic response, activate endothelial cells, which lead to endothelial damage in sepsis [5–8].

The endothelium forms a barrier that selectively controls the delivery of solutes, proteins, and cells [9], and LPS-induced endothelial hyperpermeability is a major cause of sepsis [10]. Tight junctions (TJ) are intercellular junction complexes that are crucial for epithelial and endothelial barrier function in various tissues and organisms [11]. TJ proteins, such as Zonula occluden-1 (ZO-1), occludin, and claudin-4, are critical for the maintenance of the barrier function [12–16]. Decreased expression of ZO-1 and occludin was observed in human vascular endothelial cells treated with LPS [17]. However, the molecular mechanisms that regulate this process are not fully understood.

CXC chemokine ligand 13 (CXCL13), also called BLC/BAC1, is a member of the chemokine family. It has been reported that CXCL13 levels in the serum were significantly elevated in patients with sepsis compared with that in healthy controls [18]. CXCL13 inhibits fibroblast growth factor (FGF)-2-induced chemotaxis, proliferation, and survival of human umbilical vein endothelial cells (HUVECs) [19]. CXCL13 also increased p38 phosphorylation through its sole receptor, CXC chemokine receptor 5 (CXCR5), which contributed to inflammatory pain in the dorsal root ganglia [20] and neuropathic pain in the orofacial region [21]. While CXCR5 expression has been shown in HUVECs [19], whether CXCL13 and CXCR5 are involved in LPS-induced endothelium hyperpermeability remains unknown.

In the current study, we aimed to investigate the role of CXCL13 in LPS-induced permeability of HUVECs, examine the phosphorylation of p38 during this process, and test the effects of a CXCL13-neutralizing antibody on an LPS-induced sepsis mouse model.

## MATERIALS AND METHODS

### Patients

Forty patients with sepsis (age, 24–55 years, Table 1) and 40 healthy volunteers (age, 21–53 years) were recruited at Hangzhou First People’s Hospital. The exclusion criteria included ages younger than 18 years or older than 70 years, pregnancy or neoplasm. All participants signed a written informed consent form.

**Table 1:** Demographic Characteristics of Patients with Sepsis

Variable	All	%
Age (years)	24–55	
Gender		
Male	26	65.0%
Female	14	35.0%
Smoking status		
Smoker	9	22.5%
None-smoker	31	77.5%
Alcohol abuse		
Yes	5	12.5%
No	35	87.5%

### Cell Culture and Treatment

HUVECs from the American Type Culture Collection (Rockville, MD, USA) were maintained in a humidified incubator at 37 °C, 5% CO_2_ with Dulbecco’s modified Eagle medium (Hyclone, Logan, UT, USA) containing 10% fetal bovine serum (Gibco, Carlsbad, CA, USA).

HUVECs were exposed to a series of LPS solutions (0, 50, 100, and 200 ng/ml; Solarbio, Beijing, China) for 24 h or treated with 100 ng/ml LPS for 0, 12, 24, and 48 h. The release of CXCL13, as well as the mRNA and protein level of CXCL13/CXCR5, was assessed by enzyme-linked immunosorbent assay (ELISA), real-time PCR, and western blotting, respectively.

HUVECs were transfected with CXCL13 siRNA (siCXCL13#1, 5′-GGUGUUCUGGAGGUCUAUU-3′; siCXCL13#2, 5′-CCAAGAGAGCUCAGUCUUU-3′; and siCXCL13#3, 5′-GGAAGAAGAACAAGUCAAU-3′) or control siRNA (siNC) for 24 h and then treated with 100 ng/ml LPS for 24 h; HUVECs were treated with a p38 inhibitor SB203580 (20 μM; Selleck Chemicals, Houston, TX, USA) or vehicle (DMSO) in the presence of 100 ng/ml CXCL13 (Abcam, Cambridge, MA, USA). Transendothelial electrical resistance (TER) assay and fluorescein isothiocyanate (FITC)-dextran assay were performed to evaluate cell permeability.

### Elisa

CXCL13 concentrations in the serum samples or cell culture medium were determined by ELISA kit (R&D Systems, Minneapolis, MN) according to the manufacturer’s guidelines.

### Real-Time PCR

Total RNA was prepared from HUVECs with Trizol (Invitrogen, Carlsbad, CA, USA), and reverse transcription was conducted with reverse transcription kit (Thermo Fisher Scientific, Rockford, IL, USA) following the manufacturers’ protocols. The resulted cDNA was then used as the template for real-time PCR analysis on an ABI 7300 instrument (Applied Biosystem, Foster City, CA, USA) with SYBR Green Mix (Thermo Fisher Scientific). The primers were as follows: CXCL13, 5′-GTCTGGAAGAAGAACAAG-3′ and 5′-TCTTAAACACTGGAACTG-3′; CXCR5, 5′- TCACCCTACCACATCGTCAT-3′ and 5′- CGACAGGTCACTGCGGAAC-3′; GAPDH, 5′- AATCCCATCACCATCTTC-3′ and 5′-AGGCTGTTGTCATACTTC-3′. CXCL13 and CXCR5 expressions were normalized to the internal control expression of GAPDH.

### Western Blotting

Cell lysates were prepared with RIPA buffer supplemented with protease inhibitor cocktail (Beyotime, Shanghai, China). An equal amount of protein from each sample was loaded onto 10% or 15% SDS-PAGE gel and then blotted onto nitrocellulose membranes. The membranes were blocked with 5% skim milk and then probed with primary antibodies at 4 °C overnight. After incubation with horseradish peroxidase-labeled secondary antibody (Beyotime) for 1 h at room temperature, the membranes were developed with enhanced chemiluminescence reagents (Thermo Fisher). The sources of antibodies were as follows: antibodies against CXCL13, CXCR5, ZO-1, occludin, and claudin-4 were purchased from Abcam; antibodies against p38, phosphorylated p38 (p-p38), and GAPDH were obtained from Cell Signaling Technology (Danvers, MA, USA). GAPDH levels were used as the loading control.

### TER Assay

HUVECs (1 × 10^4^ cells per well) were plated onto Transwell filters (Costar, Corning, NY, USA) and cultured at 37 °C, 5% CO_2_. After the cells formed monolayers, they were treated as indicated in figure legends. Millicell-ERS2 Volt-Ohm Meter (Millipore, Bedford, MA, USA) with electrodes was used to assess TER following the manufacturer’s protocol. TER values (Ω·cm2) were calculated by subtracting the resistance of the blank filter and correcting for the surface area (0.6 cm^2^).

### FITC-Dextran Assay

HUVECs were plated and treated as described in the TER assay. Before treatment and at 24 h after treatment, FITC-conjugated dextran (1 mg/ml, Mr. 40,000; Sigma-Aldrich) was added to the top compartment. After 2 h of culture, 100 μl samples were collected from the basal compartments and measured with a spectrofluorometer.

### Animal Experiments

All animal experiments were performed in accordance with procedures approved by the Animal Care Committee of Hangzhou First People’s Hospital. Six-week-old male C57B6/L mice (Shanghai Experimental Animal Center (Shanghai, China)) were divided into two groups (*n* = 10 per group): sepsis and control. Mice in the sepsis group and control group were administered intraperitoneally with 5 mg of LPS in 0.2 ml of saline (Solarbio) and 0.2 ml of saline, respectively. Serum concentrations of CXCL13 were determined at 24, 48, and 72 h after injection.

To study the effects of the CXCL13-neutralizing antibody, male C57B6/L mice were divided into three groups (*n* = 20 per group). Mice were treated with saline (control), LPS, or LPS plus anti-CXCL13 (Abnova, Shanghai, China; 50 μg, at 24 h before LPS treatment). The survival of mice was monitored in every 12 h for 72 h.

### Statistical Analysis

Significance was determined by a Student’s *t* test for comparison between two groups and by one-way analysis of variance (ANOVA) followed by Tukey’s test for multiple comparisons using GraphPad Prism software (GraphPad, San Diego, CA, USA). A *P* value of less than 0.05 is defined as statistically significant.

## RESULTS

### Increased CXCL13 Level Detected in the Serum of Patients with Sepsis

**Fig. 1:**
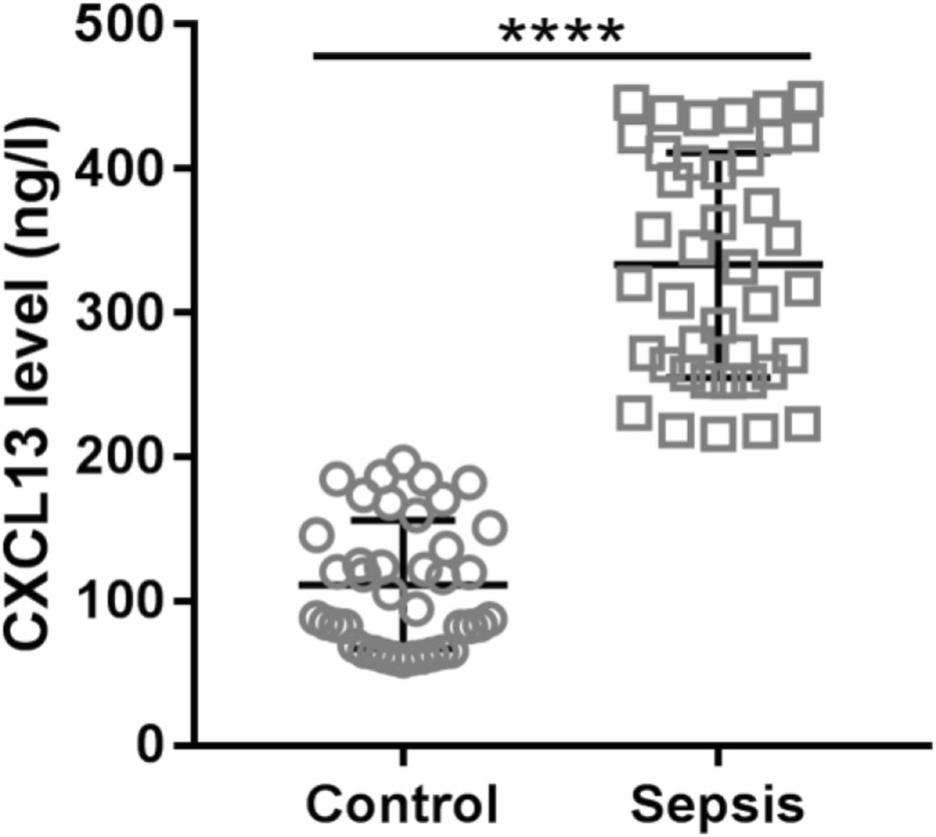
CXCL13 level was increased in the serum of patients with sepsis as indicated by ELISA assay. ^****^P < 0.0001.

ELISA analysis showed that CXCL13 was significantly elevated in the serum of patients with sepsis (*n* = 40) compared with age-matched healthy control (Fig. 1). This result provided a hint that CXCL13 might be involved in the pathology of sepsis.

### The Expression of CXCL13/CXCR5 Was Increased by LPS Exposure in a Dose- and Time-Dependent Manner

**Fig. 2:**
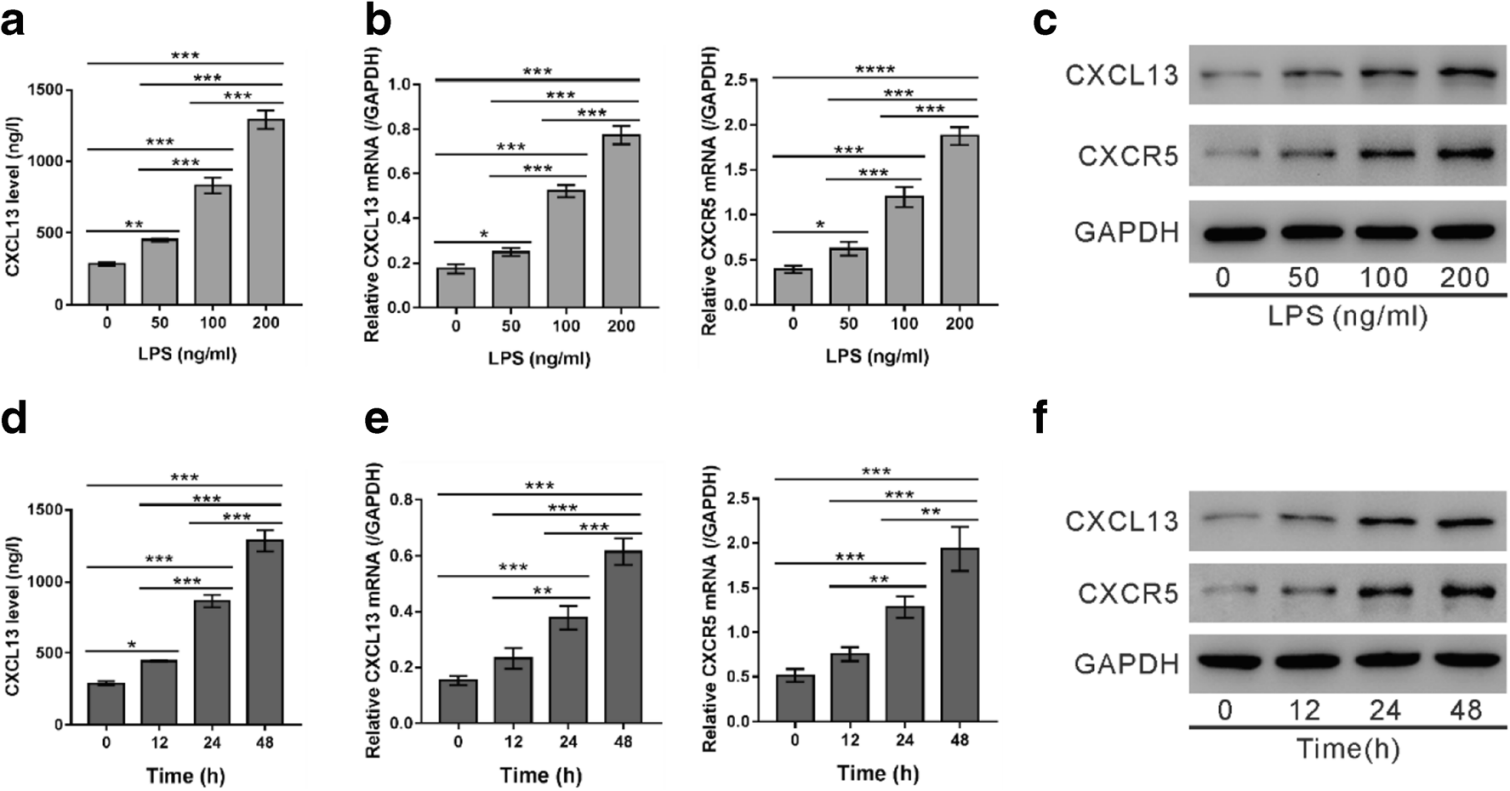
The expression of CXCL13/CXCR5 was increased by LPS exposure in a dose- and time-dependent manner. (**A**–**C**) HUVECs were treated with a series of LPS solution (0, 50, 100, and 200 ng/ml). After 24 h of exposure, ELISA (**A**), real-time PCR (**B**), and western blotting (**C**) were performed. (**D**–**F**) HUVECs were exposed to 100 ng/ml LPS for 0, 12, 24, and 48 h. ELISA (**D**), RT-PCR (**E**), and western blotting (**F**) were performed. ^*^P < 0.05, ^**^P < 0.01, ^***^P < 0.001, ^****^P < 0.0001.

HUVECs were treated with a series of LPS solution for 24 h. The release of CXCL13 (Fig. 2A), as well as mRNA (Fig. 2B) and protein (Fig. 2C) levels of CXCL13/CXCR5, was elevated by LPS treatment in a dose-dependent manner, at concentrations between 50 and 200 ng/ml.

HUVECs were then treated with 100 ng/ml LPS solution for 12, 24, and 48 h. The results showed that LPS exposure increased the release of CXCL13 (Fig. 2D), as well as mRNA (Fig. 2E) and protein (Fig. 2F) levels of CXCL13/CXCR5 in a time-dependent manner.

### CXCL13 Knockdown Abolished the Functions of LPS on HUVECs

**Fig. 3:**
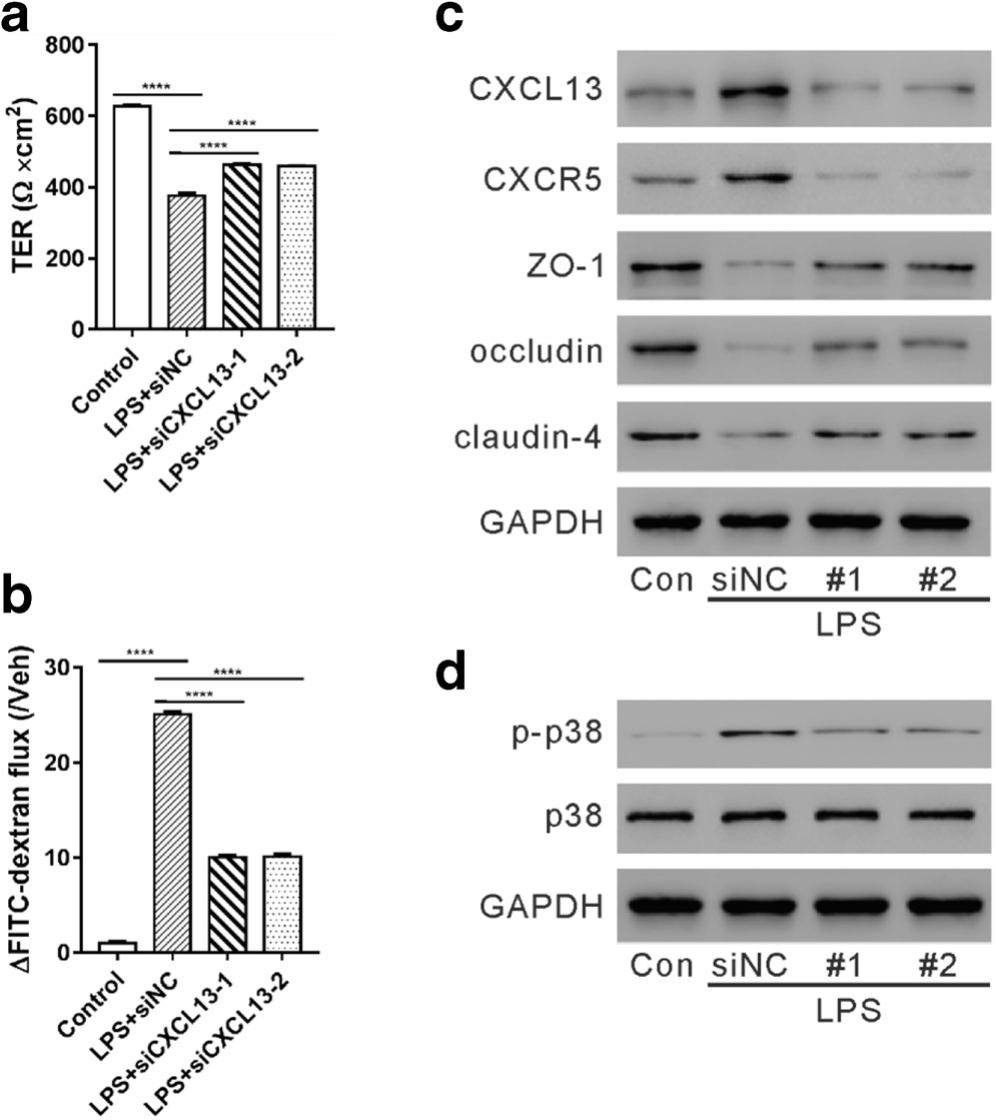
CXCL13 knockdown partially blocked the effects of LPS on HUVECs. HUVECs were transfected with CXCL13 siRNAs (siCXCL13#1, #2) or control siRNA (siNC) and then treated with 100 ng/ml LPS. Control cells were left untreated. TER assay (**A**), FITC-dextran assay (**B**), and western blotting (**C**, **D**) were performed to detect cell permeability and protein expression, respectively. ^****^P < 0.0001.

We then explored whether CXCL13 mediated LPS-induced endothelial cell permeability. First, we knocked down CXCL13 expression by transfecting the cells with CXCL13 siRNA (siCXCL13#1, #2, and #3). As shown in Fig. S1, siCXCL13#1 and #2 efficiently knocked down the protein expression of CXCL13 in HUVECs. Next, we transfected HUVECs with siCXCL13#1, siCXCL13#2, or control siRNA (siNC) and exposed the cells to LPS solution (100 ng/ml) for 24 h. LPS significantly decreased TER (Fig. 3A) and increased FITC-dextran flux (Fig. 3B). When CXCL13 expression was downregulated, the effects of LPS were partially reversed. TJ protein expression was then detected by western blotting experiments. Consistent with the results of cell permeability, expressions of ZO-1, occludin, and claudin-4 were markedly attenuated by LPS exposure, which was blocked by CXCL13 knockdown (Fig. 3C).

In addition, p38 phosphorylation was enhanced by LPS treatment as seen in previous reports [22–24]. CXCL13 knockdown, on the other hand, suppressed p38 phosphorylation caused by LPS (Fig. 3D). These data indicate that the effects of LPS on endothelial barrier function and p38 phosphorylation are partially dependent on CXCL13.

### p38 Inhibitor Partially Blocked the Functions of CXCL13 on Endothelium Hyperpermeability

**Fig. 4:**
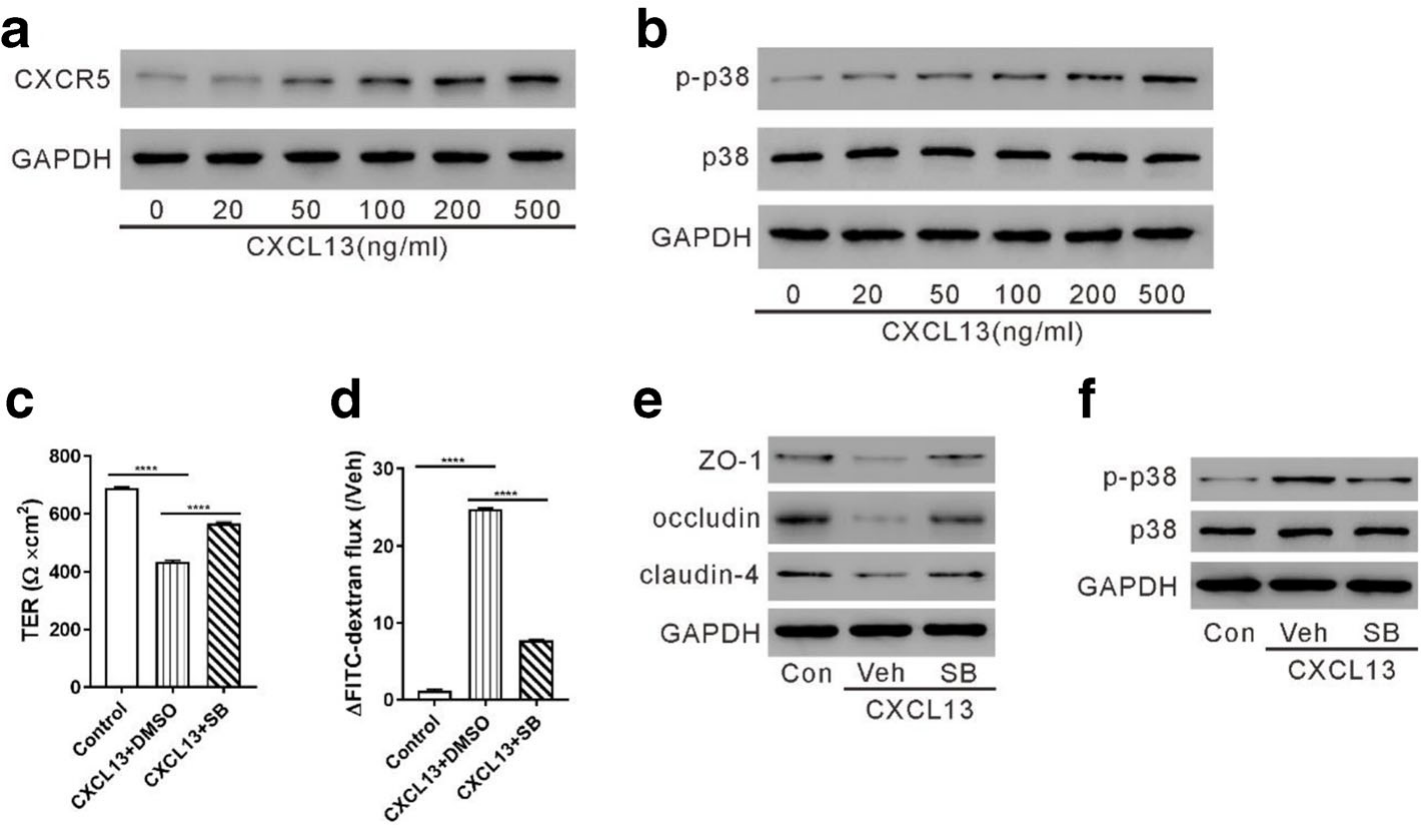
p38 partially mediated the functions of CXCL13 on endothelial cell permeability. (**A**, **B**) HUVECs were treated with a series of recombinant CXCL13 for 24 h. Western blotting was performed to detect CXCR5 and phosphorylated p38. (**C**–**F**) HUVECs were incubated with 100 ng/mL CXCL13 in the presence of SB203580 (20 μM) or DMSO. Control cells were left untreated. TER assay (**C**), FITC-dextran assay (**D**), and western blotting (**E**, **F**) were performed to detect cell permeability and protein expression, respectively. ^****^P < 0.0001.

HUVECs were treated with various concentrations of recombinant CXCL13. As illustrated in Fig. 4 A and B, CXCL13 enhanced the levels of phosphorylated p38 and CXCR5 at a concentration between 50 and 500 ng/ml. These data demonstrate the regulatory function of CXCL13 on p38 phosphorylation.

To investigate whether the p38 signaling is involved in the CXCL13-mediated action, HUVECs were exposed to a p38 inhibitor, SB203580, and then to CXCL13 (100 ng/ml). CXCL13 also increased cell permeability as indicated by the data from TER (Fig. 4C) and FITC-dextran assays (Fig. 4D). As expected, western blotting results show that CXCL13 reduced TJ protein expression and promoted p38 phosphorylation (Fig. 4E, F); these effects were partially reversed by SB203580. Together, our data imply that p38 partially mediates the functions of CXCL13 on endothelial cell permeability.

### CXCL13-Neutralizing Antibody Increased the Survival Rate of Mice with LPS-Induced Sepsis

**Fig. 5:**
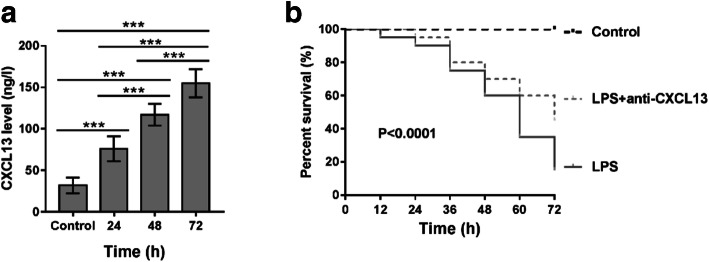
CXCL13-neutralizing antibody increased the survival rate of mice with LPS-induced sepsis. (**A**) An LPS-induced sepsis mouse model was established by injection of LPS (5 mg/kg, *n* = 10). Mice in the control group mice were injected with saline (*n* = 10). At 24, 48, and 72 h after injection, CXCL13 levels were detected by ELISA assay. ^***^P < 0.001. (**B**) Effect of CXCL13-neutralizing antibody (anti-CXCL13) on the survival rate of mice treated with LPS. Different groups (*n* = 20) of mice were treated with saline (control), LPS, or LPS plus anti-CXCL13 (50 μg at 24 h before LPS treatment). The survival rate was measured in every 12 h for 72 h.

To test the effect of CXCL13 *in vivo*, we established an LPS-induced sepsis mouse model. The administration of LPS significantly elevated the serum levels of CXCL13 in a time-dependent manner compared with that of the control mice (Fig. 5A). Furthermore, LPS injection resulted in a survival rate of less than 20% after 72 h, while the survival rate raised to approximately 40% in mice treated with the CXCL13-neutralizing antibody before LPS treatment. The differences we observed between the sepsis mice and the control mice were statistically significant.

## DISCUSSION

Several chemokines have been identified as biomarkers for sepsis, such as CXCL8, CXCL13, CCL2, and CCL8 [18, 25–28]. Here, we show that CXCL13 concentration is elevated in the serum of patients with sepsis (Fig. 1), as shown previously [18]. Elevated CXCL13 was also observed in the LPS-induced sepsis mouse model (Fig. 5) and LPS-treated HUVECs (Fig. 2). These data suggest the possible involvement of CXCL13 in the pathophysiology of sepsis. In addition, studies have shown that CXCL13 was expressed in multiple cell types, including monocytes, macrophages, dendritic cells, and endothelial cells [29, 30]. The elevation of CXCL13 in sepsis may derived from multiple cell types.

LPS-induced endothelial hyperpermeability, mediated by the regulation of the TJ protein, is a major cause of sepsis [10, 17]. Previous studies have shown that several chemokines play a critical role of regulating barrier function. For example, CXCL8 destroyed the integrity of the blood-brain barrier model *in vitro* [31], and CCL2 increased vascular permeability of HUVECs and disrupted the cellular membrane distribution of ZO-1 [32]. Here, our results from TER assay and FITC-dextran assay showed that LPS (Fig. 3) or recombinant CXCL13 (Fig. 4) exposure significantly increased endothelial cell permeability. Consistent with these results, TJ protein expression was reduced. Moreover, CXCL13 knockdown partially reversed the effects of LPS on endothelium hyperpermeability and TJ protein expression (Fig. 3). While a previous study has demonstrated the functions of CXCL13 on FGF2-induced chemotaxis, proliferation, and survival of HUVECs [19], our results give us a deeper understanding of the functions CXCL13 in endothelial cells. More importantly, pretreatment with CXCL13-neutralizing antibody significantly improved the survival rate of LPS-induced sepsis mice (Fig. 5B). Our *in vitro* and *in vivo* data, therefore, suggest that therapeutically targeting CXCL13 may be beneficial for the clinical treatment of sepsis.

P38, a serine/threonine protein kinase, belongs to the mitogen-activated protein kinase (MAPK) family. P38 is activated by environmental and cellular stress and is involved in a variety of cellular processes, such as inflammation response and cell survival [33, 34]. The levels of phosphorylated p38 were increased by LPS exposure in HUVECs [22–24]. It has been also reported that CXCL13 acts on CXCR5 and increases p38 phosphorylation during nerve injury [20, 21]. In the current study, treatment with LPS increased p-p38, which was partially reversed by CXCL13 knockdown (Fig. 3). Exposure to CXCL13 augmented p-p38, and the changes mediated by CXCL13 on cell permeability and TJ protein expression were significantly reversed by the addition of a p38 inhibitor (Fig. 4). These results suggest that the p38 signaling mediates the actions of CXCL13/CXCR5 in endothelial hyperpermeability.

In summary, serum concentrations of CXCL13 were elevated in patients with sepsis patients and in the sepsis mouse model. CXCL13/CXCR5 was upregulated by LPS exposure in HUVECs in a dose- and time-dependent manner. CXCL13 knockdown protected HUVECs from LPS-induced hyperpermeability *via* regulating the p38 pathway. Therapeutically targeting CXCL13, thus, may prove beneficial for reducing endothelial permeability and could be an effective treatment option against sepsis.
